# Dual ligation using a gastrointestinal endoscope under local anesthesia for refractory pneumothorax

**DOI:** 10.1055/a-2971-5284

**Published:** 2026-10-12

**Authors:** Ding Wang, Jisong Zhang, Yanyong Deng, Zhoumiao Chen, Shaohua Xu, Enguo Chen

**Affiliations:** 1 Department of Pulmonary and Critical Care Medicine, Regional Medical Center for National Institute of Respiratory Disease 56660 Sir Run Run Shaw Hospital, School of Medicine, Zhejiang UniversityHangzhouChina; 2 Department of Gastroenterology 56660 Sir Run Run Shaw Hospital, Zhejiang University School of MedicineHangzhouChina; 3 Department of Thoracic Surgery 56660 Sir Run Run Shaw Hospital, Zhejiang University School of MedicineHangzhouChina

Refractory pneumothorax caused by a persistent pulmonary air leak is challenging in high-risk surgical patients. 1​ ​2​ Medical thoracoscopy provides pleural access under local anesthesia and sedation. 3​ Through this access, we introduced a gastrointestinal endoscope compatible with standard ligation devices and performed dual ligation of the leaking pulmonary bulla.

A 70-year-old man with multiple bilateral pulmonary bullae developed respiratory failure and shock following rupture of a left-sided bulla. At a local hospital, he underwent emergency endotracheal intubation, resuscitation, and chest-tube drainage. After several weaning attempts, he was eventually extubated and later referred to our institution for refractory left pneumothorax. On arrival, he was clinically stable, but persistent air leakage had required chest-tube drainage for over 1 month. His medical history included right lower lobectomy and systemic antitumor therapy. Given his limited pulmonary reserve, thoracic surgeons considered video-assisted thoracoscopic surgery under general anesthesia and single-lung ventilation prohibitively risky. After multidisciplinary discussion and shared decision-making, medical thoracoscopy was selected.

Although the prolonged air leak impaired his quality of life, the pneumothorax provided a natural intrapleural working space. Under local anesthesia and sedation, standard medical thoracoscopic access was established by blunt chest-wall dissection and trocar insertion. An ethylene oxide-sterilized flexible gastrointestinal endoscope, selected for compatibility with standard ligation accessories, was fitted with a multiband ligator and advanced through the trocar. Direct inspection and saline testing revealed bubbling from the target bulla, confirming the air-leak site ( Fig. 1a ). The bulla was suctioned into the ligator cap ( Fig. 1b and Video 1 ), banded at its base ( Fig. 1c ), and deflated ( Fig. 1d ). To prevent band slippage as negative pleural pressure was restored during lung re-expansion, an endoscopic ligation loop was advanced through the working channel and tightened on the lung side of the elastic band ( Fig. 1e, f ). Repeat saline testing showed no bubbling.

**Fig. 1:**
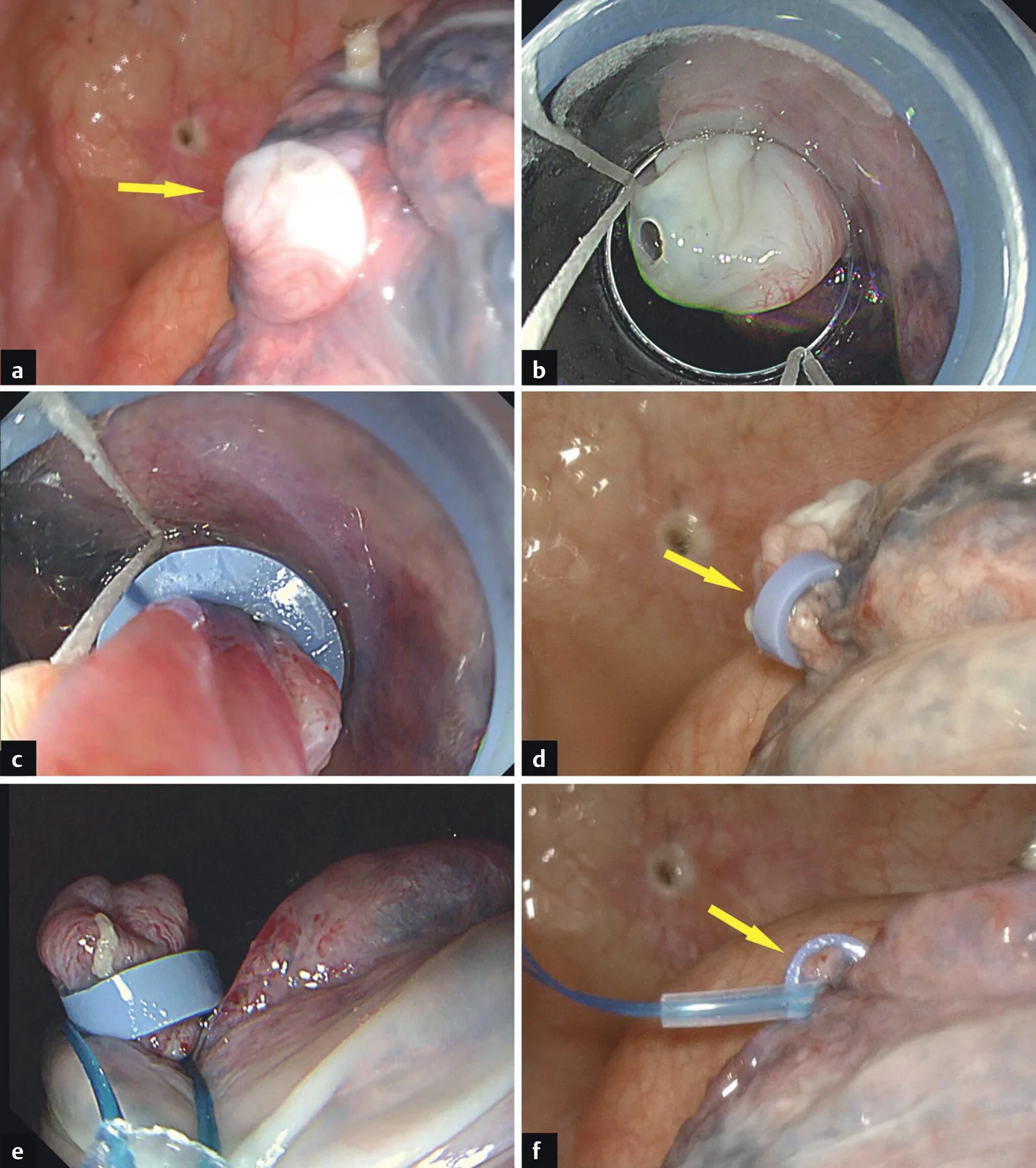
Gastrointestinal endoscopic dual ligation of a leaking pulmonary bulla. Panel **a** shows the intrapleural endoscopic view, identifying the target bulla and the air-leak point (yellow arrow). Panel **b** shows the bulla being aspirated into the multi-band ligator. Panel **c** shows the deployment of an elastic band at the base of the aspirated bulla. Panel **d** shows the deflated bulla with the elastic band secured at its base (yellow arrow). Panel **e** shows a dedicated endoscopic ligation loop introduced through the endoscope’s working channel to reinforce the ligation below the elastic band. Panel **f** shows the final appearance of the dual-ligated bulla (yellow arrow).

**Video 1** The leaking pulmonary bulla was suctioned into the ligator cap and secured with an elastic band; a nylon ligation loop was then tightened on the lung side of the elastic band to reinforce the ligation.


On postoperative day 4, chest radiography confirmed the complete resolution of the pneumothorax and full re-expansion of the left lung. The chest tube was subsequently removed, and the patient was discharged without complications. At 1 month, computed tomography showed the disappearance of the target bulla without recurrent pneumothorax ( Fig. 2 ). Continued clinical and imaging follow-up was planned to evaluate the durability of air-leak closure and the long-term safety of the retained elastic band and ligation loop.

**Fig. 2:**
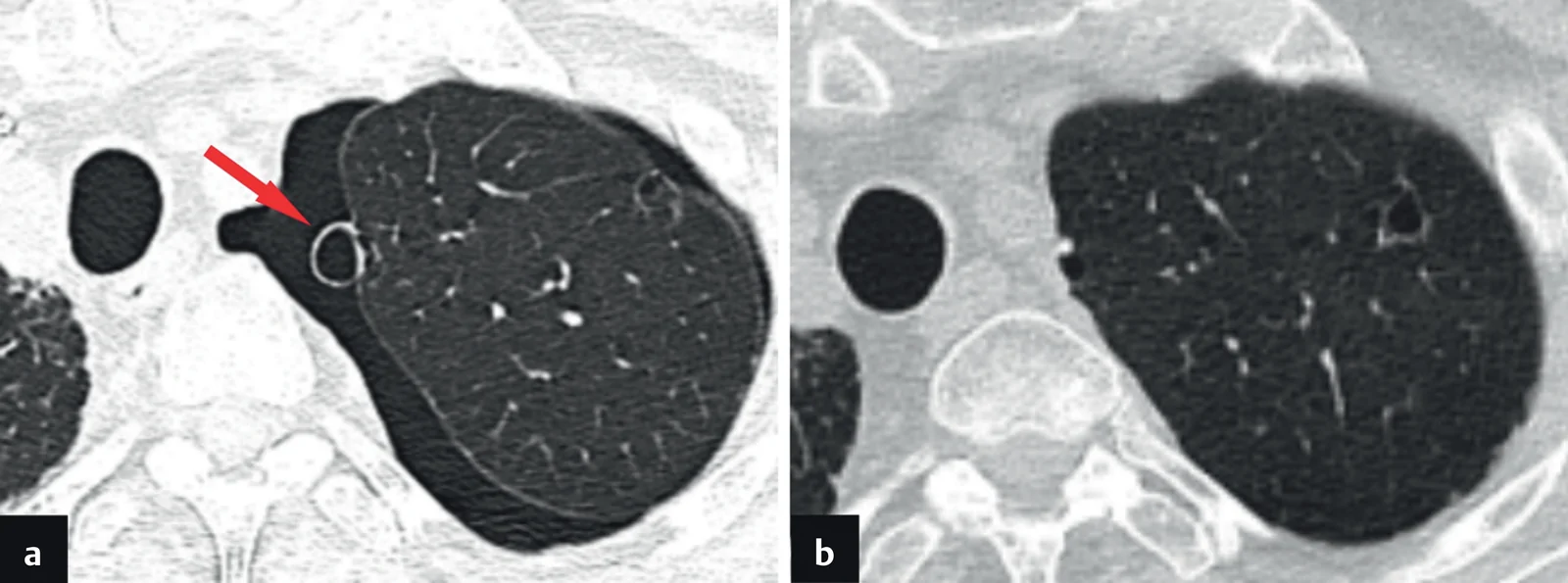
A preoperative chest CT scan (Panel a) shows the target bulla (red arrow). At 1-month follow-up, the postoperative CT scan (Panel b) shows the complete disappearance of the target bulla and full resolution of the pneumothorax. CT, computed tomography.

This technique may provide a nonresectional alternative for selected patients with a localized pulmonary bulla, persistent air leak, and prohibitive surgical risk.

Endoscopy_UCTN_Code_TTT_1AO_2AO

## Declaration of GenAI Use

The authors confirm that no generative artificial intelligence was used in the preparation of this manuscript.
